# Psoas Muscle Volume Is a Predictive Marker of Fatigue and Anorexia in Prostate Cancer Patients Treated with Enzalutamide

**DOI:** 10.31662/jmaj.2022-0096

**Published:** 2022-09-20

**Authors:** Eiji Kashiwagi, Masaki Shiota, Ken Lee, Keisuke Monji, Hidekazu Naganuma, Takashi Matsumoto, Ario Takeuchi, Junichi Inokuchi, Masatoshi Eto

**Affiliations:** 1 Department of Urology, Graduate School of Medical Science, Kyushu University, Fukuoka, Japan

**Keywords:** psoas muscle, prostate cancer, enzalutamide, body composition, fatigue, anorexia

## Abstract

**Introduction:**

Enzalutamide is approved for the treatment of patients with metastatic castration-resistant prostate cancer. Adverse effects (e.g., fatigue and anorexia) are often observed and cause difficulty with continuous therapy; however, no clinical data describing which patients are more likely to suffer adverse effects were observed. Therefore, this study hypothesized that body composition, comprising body fat distribution and psoas muscle volume, may affect the occurrence of subjective symptoms (e.g., fatigue and anorexia) in prostate cancer patients treated with enzalutamide.

**Methods:**

Adverse effects, especially fatigue, anorexia, insomnia, and pain, were retrospectively evaluated by CTCAE v4.0 criteria. Sixty-seven prostate cancer patients treated with enzalutamide were enrolled, and body fat, visceral fat percentage, and psoas muscle ratio (psoas muscle, in cubic centimeter/height, in meters) were calculated using computed tomography images evaluated before enzalutamide, with SYNAPSE VINCENT software. Univariate analysis was performed to identify the factors associated with adverse effects.

**Results:**

Univariate analysis showed that high psoas muscle ratio was significantly associated with fatigue (grade ≥ 2; odds ratio, 3.875; 95% confidence interval, 1.016-17.134; *P* = 0.047), but inversely related to anorexia (grade ≥ 2; odds ratio, 0.093; 95% confidence interval, 0.011-0.784; *P* = 0.029).

**Conclusions:**

Psoas muscle ratio is a predictive marker of fatigue and anorexia in patients treated with enzalutamide.

## Introduction

Prostate cancer (PCa) is one of the most common noncutaneous cancers in men in developed countries ^(1), (2)^. Androgen deprivation therapy (ADT) has been the gold standard for metastatic PCa because ADT suppresses testosterone production ^(3)^. Most PCas initially respond well to ADT but eventually relapse in a castration-resistant manner and are then defined as castration-resistant prostate cancer (CRPC) ^(4)^.

Enzalutamide is designed to exert its antitumor activity by binding to androgen receptors (AR) of the ligand-binding domain and prevents AR translocation to the cell nucleus, recruitment of AR cofactors, and DNA binding ^(5)^. Enzalutamide showed good overall survival and radiographic progression-free survival in CRPC patients ^(6), (7)^, but adverse effects (e.g., fatigue) are commonly reported adverse events that must be addressed. Therefore, determining the characteristics of patients who are more likely to be affected by adverse events associated with enzalutamide is important.

In this study, body composition (e.g., body fat distribution and psoas muscle volume) was hypothesized to affect subjective symptoms (e.g., fatigue and anorexia), caused by enzalutamide, and the relationship between body composition and these symptoms were investigated.

## Materials and Methods

### Study design

This was a retrospective study that enrolled patients with CRPC who were treated with enzalutamide at Kyushu University Hospital (Fukuoka, Japan) from August 2011 to September 2018. This study (# 2021-123) was approved by the hospital’s institutional review board. All patients were histopathologically verified with adenocarcinoma of the prostate and were primarily treated by ADT with medical castration using a luteinizing hormone-releasing hormone (LHRH) agonist (leuprorelin acetate or goserelin acetate) or LHRH antagonist (degarelix). Adverse effects (e.g., fatigue, anorexia, pain, and insomnia) were retrospectively assessed using the CTCAE v4.0 criteria by chart review. In this study, fatigue, anorexia, and pain were defined as positive for grade ≥2 toxicity, and insomnia was defined as positive for grade ≥1 toxicity.

Computed tomography (CT) images were examined before enzalutamide administration, and visceral fat volume, subcutaneous fat volume, psoas muscle volume, and abdominal volume were calculated using SYNAPSE VINCENT software (Fuji Film, Tokyo, Japan) as previously described ^(8)^. Briefly, adipose tissue was identified as pixels ranging from −250 to −50 Hounsfield units. The visceral fat volume was divided by the subcutaneous fat volume to calculate the visceral fat/subcutaneous fat ratio (V/S ratio). The psoas muscle volume (in cubic centimeters) was divided by the patient’s height (in meters) to calculate the psoas muscle ratio. Abdominal volume and subcutaneous fat were calculated from the diaphragm to the pubic bone level.

### Statistical analysis

All statistical analyses were performed using JMP14 software (SAS Institute Inc., Cary, NC, USA). Univariate analysis was performed using a logistic regression model. Correlations between parameters were examined by the *χ*^2^ test, and *P* values of <0.05 were considered significant.

## Results

### Univariate analysis of the relationship between the patients’ characteristics and adverse effects

The patients’ characteristics are summarized in Table 1. Eighteen (26.8%) patients received corticosteroids, and 23 (34.3%) had received treatment with docetaxel before enzalutamide administration.

**Table 1.** Patients’ Characteristics (*n* = 67).

Variable		
Median age, years (range)		73 (47-89)
Biopsy Gleason score, *n* (%)		
	<8	16 (23.8%)
	≥8	50 (73.6%)
	NA	1 (1.4%)
Median PSA at pretreatment, ng/mL (range)		14.2 (0.224-8214)
Clinical stage at diagnosis, *n* (%)		
	N0M0	31 (46.2%)
	N1M0	4 (5.9%)
	M1	32 (47.7%)
Prior docetaxel		23 (34.3%)
Prior abiraterone		8 (11.9%)
Corticosteroid administration		18 (26.8%)
Median psoas muscle ratio, cm^3^/m (range)		160.1 (87.3-240.1)
Median visceral fat, % (range)		36.4 (11.1-58.3)
Median body fat, % (range)		34.5 (12.3-51.5)
Median V/S ratio, ratio (range)		1.04 (0.46-2.12)
Fatigue (grade^a,b^)		
	0	36 (53.7%)
	1	19 (28.3%)
	2	10 (14.9%)
	3	2 (2.9%)
Anorexia (grade)		
	0	44 (65.6%)
	1	13 (19.4%)
	2	7 (10.4%)
	3	3 (4.4%)
	4	0
	5	0
Pain (grade)		
	0	54 (80.5%)
	1	6 (8.9%)
	2	2 (2.9%)
	3	5 (7.4%)
Insomnia (grade)		
	0	63 (94.0%)
	1	3 (4.4%)
	2	1 (1.4%)
	3	0

^a^Fatigue, anorexia, pain, and insomnia were graded following the CTCAE criteria, version 4.0. ^b^Values are number (percent) for each parameter. Psoas muscle ratio: psoas muscle volume/height (cm^3^/m) Body fat: (visceral fat + subcutaneous fat)/abdominal volume *NA* not available, *N* node, *M* metastasis, *PSA* prostate-specific antigen,* V/S ratio* visceral fat/subcutaneous fat ratio

Univariate analysis using a logistic regression model was performed to investigate the important factors influencing the adverse effects (e.g., fatigue and anorexia). Patients were divided into two groups following the median of each of the following parameters: psoas muscle ratio, body fat, visceral fat, and V/S ratio. The univariate analysis identified higher psoas muscle ratio (odds ratio [OR], 3.875; 95% confidence interval (CI), 1.016-17.134; *P* = 0.047) as an independent predictor of fatigue (Table 2). In contrast, higher psoas muscle ratio (OR, 0.093; 95% CI, 0.011-0.784; *P* = 0.029) was associated with a significantly lower incidence of anorexia.

**Table 2.** Univariate Analysis of Adverse Effects in CRPC Patients Treated with Enzalutamide. Psoas Muscle Ratio, Body Fat, and Visceral Fat Volumes, and the V/S Ratio Were Used to Divide the Patients into Two Groups Following the Median Values for Each Parameter.

Parameter	Fatigue (>G2)			Anorexia (>G2)			Pain (>G2)			Insomnia (>G1)		
	OR	95% CI	*P* value	OR	95% CI	*P* value	OR	95% CI	*P* value	OR	95% CI	*P* value
Age	1.012	0.939-1.091	0.744	1.075	0.984-1.176	0.108	0.942	0.858-1.033	0.208	1.018	0.901-1.151	0.765
Prior docetaxel												
−	1			1			1			1		
+	0.947	0.252-3.555	0.936	0.792	0.184-3.406	0.755	2.877	0.585-14.147	0.193	0.621	0.060-6.331	0.678
Corticosteroid												
−	1			1			1			1		
+	0.487	0.095-2.477	0.386	2.047	0.504-8.316	0.316	2.25	0.451-11.222	0.326	0.901	0.087-9.275	0.93
Psoas muscle ratio												
<160.1	1			1			1			1		
≥160.1	3.875	1.016-17.134	0.047	0.093	0.011-0.784	0.029	0.801	0.165-3.893	0.784	1.099	0.145-8.303	0.924
Body fat												
<34.5	1			1			1			1		
≥34.5	2.074	0.558-7.698	0.275	0.559	0.142-2.196	0.405	1.247	0.256-6.057	0.784	2.906	0.286-29.468	0.366
Visceral fat												
<36.4	1			1			1			1		
≥36.4	2.230	0.600-8.282	0.230	0.600	0.152-2.356	0.464	0.701	0.144-3.407	0.660	3.096	0.305-31.399	0.338
V/S ratio												
<1.04	1			1			1			1		
≥1.04	1.350	00381-4.776	0.641	4.444	0.866-22.783	0.073	0.327	0.058-1.821	0.202	2.906	0.286-29.468	0.366

*G* grade following the CTCAE criteria, version 4.1; *CRPC* castration-resistant prostate cancer; *OR* odds ratio; *CI* confidence interval Psoas muscle ratio: psoas muscle volume/height, (cm^3^/m); body fat: (visceral fat + subcutaneous fat)/abdominal volume; V/S ratio: visceral fat/subcutaneous fat ratio

### Psoas muscle ratio is related to age and fat

The relationship between psoas muscle ratio and each parameter was also investigated. Younger patients tended to have a higher psoas muscle ratio compared to older patients, and body fat and visceral fat volumes were larger in the higher psoas muscle ratio group than those in the lower psoas muscle ratio group (*P* = 0.028, 0.002, and 0.001, respectively; Table 3).

**Table 3.** Relationship between Psoas Muscle Ratio and Each Parameter.

Psoas muscle ratio			
Parameter	<160.1	≥160.1	*P* value
Median age (range)	72 (47-87)	68 (55-89)	0.028
Prior docetaxel	13 (19.4)	10 (14.9)	0.611
Prior abiraterone	6 (8.9)	2 (2.9)	0.169
Corticosteroid administration	10 (14.9)	8 (11.9)	0.741
Median body fat (range)	31.7 (12.3-49.8)	37.1 (22.2-51.5)	0.002
Median visceral fat (range)	31.8 (11.1-58.3)	39.9 (18.3-55.8)	0.001
Median V/S ratio (range)	0.96 (0.46-1.97)	1.15 (0.58-2.12)	0.118

Psoas muscle ratio: psoas muscle volume/height (cm^3^/m) Body fat: (visceral fat + subcutaneous fat)/abdominal volume V/S ratio: visceral fat/subcutaneous fat ratio

## Discussion

This study aims to test the hypothesis that body composition is a predictive marker of adverse events (e.g., fatigue and anorexia) with enzalutamide therapy in CRPC patients. The findings of the current study indicated that a high psoas muscle ratio was a predictive marker for a higher incidence of fatigue but a lower incidence of anorexia compared with a low psoas muscle ratio.

ADT has many adverse effects (e.g., loss of libido, erectile dysfunction, psychological distress, decreased muscle strength, obesity, suppressed physical activity, and fatigue) ^(9)^. PCa patients who have variant alleles of the interleukin-6 and tumor necrosis factor-alpha genes are sensitive to fatigue with therapy with goserelin or leuprolide ^(10)^. Additionally, more comorbidities and higher Gleason scores were reported as risk factors for ADT-related fatigue compared with patients with fewer comorbidities and lower Gleason scores ^(11)^. Fatigue is one of the major symptoms in PCa treatment; medical castration for PCa causes clinically relevant fatigue in 43% of patients ^(12)^. However, antiandrogen monotherapy, such as with bicalutamide, was reported to cause not more than a 5% incidence of fatigue ^(13)^. In metastatic PCa patients, the TERRAIN study revealed that enzalutamide with ADT induced more fatigue compared with bicalutamide with ADT (28% vs. 20%, respectively; any grade) ^(14)^. Additionally, for CRPC patients, the STRIVE trial showed that fatigue was more relevant with enzalutamide compared with bicalutamide (38% vs. 28%, respectively; any grade) ^(15)^. A meta-analysis of randomized controlled trials of metastatic CRPC patients treated with abiraterone or enzalutamide showed that abiraterone had no significant association with fatigue and that enzalutamide showed a higher incidence of fatigue compared with abiraterone (relative risk, 1.29; 95% CI, 1.16-1.44; *P* < 0.0001) ^(16)^. Patients treated with enzalutamide were forced to have a dose reduction ^(17)^, which may worsen the prostate-specific antigen response, because of fatigue ^(18)^.

An association between cancer-related fatigue and muscle strength and/or muscle volume was linked in some reports ^(19), (20)^, and performance status and psychological distress are also related to fatigue ^(21)^. Fatigue is a subjective experience and is caused by a general response to physical or psychological stress ^(22)^. Rest and/or relaxation are refreshing for healthy individuals, but fatigue continues chronically and limits the daily activities of some cancer patients ^(22)^. Generally, the mechanisms of fatigue are divided into two groups, central and peripheral ^(23)^. Central fatigue may derive from the cerebral cortex and/or the spinal cord. Serotonin (5-HT) concentration and/or 5-HT receptor activation in the brain are also involved in cancer-related fatigue ^(24)^. Testosterone was suggested to modulate 5-HT receptor expression in the rat brain ^(25)^, and serotonin modulates AR expression in the rat prostate ^(26)^. This study also revealed that the 5-HT receptor is a modulator of AR in CRPC cell lines ^(27)^. Furthermore, bicalutamide suppressed the expression of 5-HT and induced depression in rats ^(28)^. These results suggest that AR and 5-HT have a close relationship such that AR inhibitors (e.g., enzalutamide) may cause fatigue owing to 5-HT modulation. Enzalutamide showed a five-times higher affinity for AR compared with bicalutamide ^(29)^, which may induce fatigue more frequently.

In contrast to central fatigue, peripheral fatigue may arise from muscle. ARs are also present in skeletal muscle, and AR antagonists affect muscle and prevent muscle hypertrophy ^(30), (31)^. Enzalutamide works not only in the prostate but also in muscle and may cause peripheral fatigue by affecting AR signaling. The results of the current study showed that psoas muscle volume was related to fatigue incidence. Patients with more muscle mass may have more AR expression throughout the body and may feel fatigued more frequently. However, several newly discovered drugs cause fatigue quite often ^(32)^, and the mechanisms of fatigue are not simple; various factors may be involved, and further studies are needed.

Anorexia is also one of the symptoms that worsen patients’ quality of life. In the AQUARiUS study, enzalutamide caused anorexia in 60% of CRPC patients ^(33)^. Among Japanese patients, enzalutamide caused anorexia in 13% of patients ^(34)^, but coadministration of corticosteroids improved the incidence of anorexia and fatigue ^(35)^. In the present study, 33.8% of the patients showed anorexia, and a lower psoas muscle ratio was an independent predictor of the incidence of anorexia (Table 2). In CRPC patients, 34.0%-74.9% were reported to have sarcopenia ^(36), (37)^. After a median of 10.8 months on enzalutamide treatment, 5.2% skeletal muscle loss was observed ^(38)^, and enzalutamide administration for 24 months increased the rate of sarcopenia from 24.1% to 39.3% ^(39)^. Lean psoas muscle volume, evaluated in this study, was associated with low body or visceral fat (Table 3) and may reflect sarcopenia. Patients with sarcopenia are easily affected by the adverse effects of chemotherapeutic drugs ^(40)^ and may develop anorexia. Additionally, skeletal muscle mass decreases as a result of anorexia, which may lead to a vicious cycle of worsening sarcopenia.

In this study, anorexia and fatigue did not correlate (data not shown), a discrepancy was observed between anorexia and fatigue regarding the psoas muscle ratio. Serum concentrations of inflammatory biomarkers and cancer-related symptoms are related, and one study showed that interleukin-6 and C-reactive protein concentrations were associated with anorexia; however, interleukin-1ra was associated only with fatigue ^(41)^. In contrast, the results of some studies are inconsistent with those of the previous study showing that C-reactive protein represents a marker of fatigue ^(42), (43)^. Furthermore, fatigue in PCa patients is suspected to involve genetic issues ^(10)^. These results suggest that identifying a predictive biomarker for enzalutamide-related adverse effects from peripheral blood is not easy, and being able to select patients who are more likely to have adverse effects, using CT, could be useful for clinicians.

Improving fatigue is important for better patient quality of life, and depression and pain independently interfere with fatigue in PCa patients receiving ADT ^(12)^. Exercise was recommended for these patients, which improved their quality of life and reduced fatigue incidence ^(44)^. Medications (e.g., methylprednisolone) are options for treating cancer-related fatigue ^(45)^. Only a few published case reports were reported regarding the treatment of adverse events associated with enzalutamide, but initiating a temporary drug holiday ^(46)^ or changing the timing of medication ^(47)^ was reportedly effective. Further studies are needed to manage fatigue to achieve better tolerance.

Although psoas muscle volume calculated using CT images appears promising, the sample size for this study was relatively small. In this study, the psoas muscle was focused on because its volume is easily calculated and it is a core muscle. In the future, the detailed mechanism by which the psoas muscle contributes endocrinologically and structurally to adverse effects (e.g., fatigue and anorexia) must be investigated.

In conclusion, compared with a lower psoas muscle ratio, a higher psoas muscle ratio was a predictive marker for a higher incidence of fatigue and a lower incidence of anorexia in CRPC patients receiving treatment with enzalutamide. Predicting the occurrence of adverse effects (e.g., fatigue and anorexia), using CT images is important when prescribing enzalutamide, and appropriate treatment should be provided.

## Article Information

### Conflicts of Interest

Masaki Shiota received honoraria from Janssen Pharmaceutical, AstraZeneca, and Astellas Pharma and research funding support from Daiichi Sankyo.

### Sources of Funding

This work was supported by a Grant-in-Aid for Young Scientists (B) from the Japan Society for the Promotion of Science (Number 16K20143).

### Acknowledgement

The authors thank Jane Charbonneau, DVM, from Edanz (https://jp.edanz.com/ac) for editing the draft of this manuscript.

### Author Contributions

Conceptualization: EK and MS. Methodology: EK, MS, and ME. Data collection and analysis: KM, KL, HN, TM, and AT. Manuscript writing: EK. Review and editing of the manuscript: SM and JI.

### Approval by Institutional Review Board (IRB)

This study (#2021-123) was approved by the Institutional Review Board of Kyushu University Hospital (Fukuoka, Japan).

### Consent to Participate and Consent to Publish

Informed consent was achieved by an opt-out approach.
